# Radiation exposure lymphocyte damage assessed by γ-H2AX level using flow cytometry

**DOI:** 10.1038/s41598-024-54986-x

**Published:** 2024-02-22

**Authors:** Zhuoqing Chen, Hiroshi Wakabayashi, Rie Kuroda, Hiroshi Mori, Tomo Hiromasa, Daiki Kayano, Seigo Kinuya

**Affiliations:** 1 Department of Nuclear Medicine, Kanazawa University Hospital, 13-1 Takara-machi, Kanazawa, Ishikawa 920-8641 Japan; 2 Department of Pediatrics, Kanazawa University Hospital, 13-1 Takara-machi, Kanazawa, Ishikawa 920-8641 Japan

**Keywords:** Cancer, Molecular medicine, Oncology, Risk factors, Signs and symptoms

## Abstract

DNA double-strand breaks (DSBs) are considered the most relevant lesions to the DNA damage of ionizing radiation (IR), and γ-H2AX foci in peripheral blood lymphocytes are regarded as an adequate marker for DSB quantitative studies. This study aimed to investigate IR-induced DNA damage in mice through γ-H2AX fluorescence analyses by flow cytometry (FCM). The levels of γ-H2AX in CD4/CD8/B220-positive lymphocytes were quantified by FCM through mean fluorescence intensity (MFI) values. Peripheral venous blood samples were collected for evaluation, and all the control groups were restrained from irradiation. For external irradiation experiments, the dose-dependency of MFI values and temporal alternations were assessed both in vitro and in vivo. External radiation exposure damage was positively correlated with the absorbed radiation dose, and the lymphocyte recovered from damage within 3 days. I-131 sodium iodide solution (74 MBq) was injected into the mice intraperitoneally for internal irradiation experiments. Gamma counting and γH2AX foci analyses were performed at 1 h and 24 h by the group. The blood-to-blood *S* values (*S*_blood←blood_) were applied for the blood-absorbed dose estimation. Internal low-dose-irradiation-induced damage was proved to recover within 24 h. The FCM method was found to be an effective way of quantitatively assessing IR-induced DNA damage.

## Introduction

Ionizing radiation (IR) is of critical importance in cancer therapy through the induction of deoxyribonucleic acid (DNA) damage, and external and internal radiation therapy are both widely used in clinical practice. However, IR has short-term to long-term adverse effects^1^ on non-neoplastic cells. Assessing the IR dose administered to cells is crucial to minimize these effects, and estimation of DNA damage induced by radiation could be valuable, for which double-strand breaks (DSBs) are considered most relevant^2^. The formation of γ-H2AX foci is known for its effectiveness in DSB detection^3,4^. H2AX is one of the three subfamilies of the H2A histone protein family that plays a crucial role in the crystallographic structure of nucleosomes, and it was elucidated that the induction of H2AX γ-phosphorylation, namely the formation of γ-H2AX foci, serves as a rapid and sensitive indicator of IR exposure-relevant DSB^5^.

Blood tests have been ubiquitously used to evaluate residual damage after whole-body irradiation^6^. Distinct from local external radiation treatment, internal radiation therapy irradiates the whole body, which suggests that the residual absorbed dose to the blood is sufficient for the estimation of whole body exposure dose. Lymphocytes, which are radiosensitive white blood cells^7^, are easily classified by a cluster of differentiation through flow cytometry (FCM). The radiosensitivity of different immunophenotype subsets has been reported. Zárybnická et al. reported radioresistance in NK cells and radiosensitivity in T and B cells 24 h after external irradiation^5^. Wilkins et al. found that CD8 + T-cells were more liable to radiation-induced apoptosis than CD4 + T-cells at doses above 2 Gy^8^. To select sensitive indicators and establish a quantitative evaluation method, γ-H2AX fluorescence analyses in lymphocytes were proposed to quantitatively assess the IR-induced damage. This study focused on radiosensitive lymphocytes, including CD4+ , CD8 + T lymphocytes, and CD45R/B220 + B lymphocytes^5,9^.

The traditional DSB study method of lymphocytes is to count γ-H2AX and 53BP1 foci through immunofluorescence microscopy^10,11^, which requires complicated protocols and much time. Besides, previous studies demonstrated that the γ-H2AX assay, in addition to the colocalization of γ-H2AX and 53BP1 foci analyses, also had the potential to assess DSB repair capacity^12–14^. Compared to immunofluorescence microscopy, FCM is relatively more accessible and capable of multiparametric data acquisition and expediting the process of comparing specific cellular subsets in an efficient manner^15^. Notably, DNA damage induced by low-dose whole-body exposure can hardly be measured through FCM, even though high-dose exposure is convincingly detectable^14^.

In the present study, we investigated residual DNA damage caused by external and internal irradiation in mice's radiosensitive lymphocyte subsets using FCM γ-H2AX fluorescence analyses.

## Results

### Immunohistochemistry staining analyses

**Figure 1:**
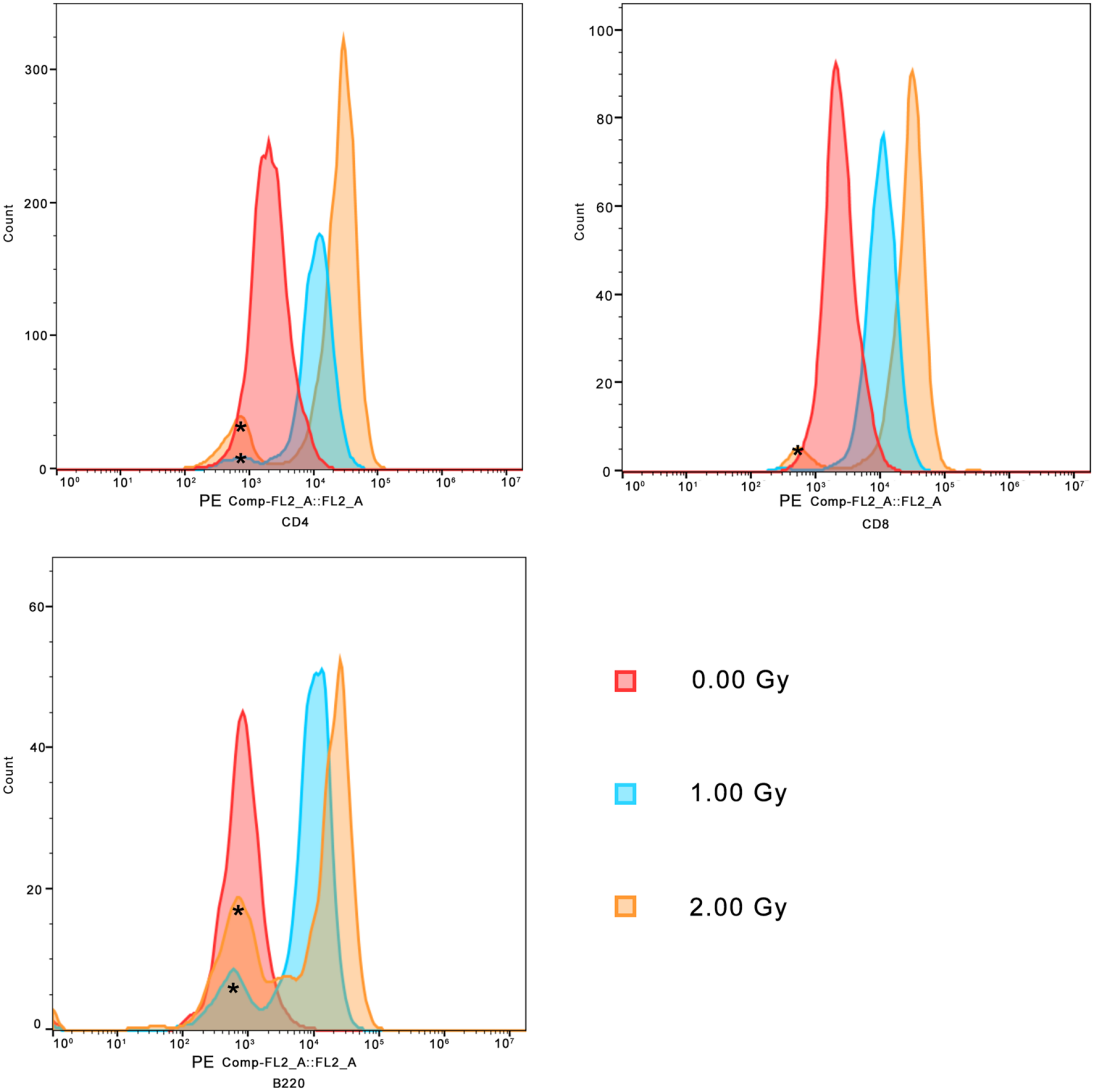
Illustration of positive dose-dependency changes in MFI values in CD4 + Lymphocytes. The y-axis represents the cells count. *Autofluorescence of unstained cells.

Mean fluorescence intensity (MFI) values were used to estimate the γ-H2AX level in FCM analysis. The degree of change is measured using a ratio of MFI values after irradiation (MFI’) and MFI values of the controls (MFI). All the IHC staining images were analyzed via Image J (Version Java 1.8.0, Rasband, W.S., ImageJ, U. S. National Institutes of Health, Bethesda, Maryland, USA), and DNA damage were quantified as a ratio of γ-H2AX cell count after irradiation and count of the nuclei. γ-H2AX cell count analyses were performed, and results are shown in Table 1. The MFI values were represented using histograms in FlowJo. Three representative examples of CD4+ , CD8+ , and B220 + lymphocytes after irradiation of 0 Gy, 1 Gy, and 2 Gy are shown in Fig. 1. It was observed that the MFI values increase with dose.

**Table 1:** t-Test results of γ-H2AX cell count analyses in external exposure experiments.

Irradiation doses (Gy)	0 Gy	1 Gy	*P*-value of ANOVA
Count ratio	0.18 ± 0.03*	0.32 ± 0.03 *	< 0.05

*0 Gy versus 1 Gy, *P* < 0.05.

### Linear positive correlation between γ-H2AX levels and doses

The in vivo and in vitro experiments of external irradiation showed that the ratio of MFI'/MFI was positively correlated with the radiation doses. The results of the linear regression analysis are shown in Table 2. Notably, B220 + cells (B-cells) represented the greatest linear correlation with DNA damage in the in vitro experiments, whereas in vivo experiment results showed that CD4 + and CD8 + lymphocytes (T-cells) had the best linear correlation.

**Table 2:** Radioactive correlation linear fit in external exposure experiments.

Cells	Linear fit equation	R^2^	Prob > F	Prob >|t|
In vitro experiments				
CD4 (+)	MFI'/MFI = 1.13 + 4.33*Radiation (Gy)	0.52	< 0.0001	< 0.0001
CD8 (+)	MFI'/MFI = 1.00 + 4.03*Radiation (Gy)	0.49	< 0.0001	< 0.0001
B220 (+)	MFI'/MFI = 0.84 + 6.36*Radiation (Gy)	0.76	< 0.0001	< 0.0001
In vivo experiments				
CD4 (+)	MFI'/MFI = 0.72 + 3.85*Radiation (Gy)	0.84	< 0.0001	< 0.0001
CD8 (+)	MFI'/MFI = 0.67 + 3.92*Radiation (Gy)	0.84	< 0.0001	< 0.0001
B220 (+)	MFI'/MFI = 0.77 + 6.06*Radiation (Gy)	0.67	< 0.0001	< 0.0001

### Temporal alternation

In the external irradiation experiments, no significant difference between the ratio of MFI’ to MFI on days 3 and 7 was observed regardless of the radiation doses; however, differences between days 0 and 3 were proved significant in the experimental groups that accepted radiation dose of more than 0.10 Gy. The t-test results are shown in Table 3.

**Table 3:** t-Test results of external exposure in in vivo experiments.

Cells	Irradiation doses (Gy)	Level	−Level	*P*-value
CD4 (+)	0.10	0 d	3 d	0.93
		3 d	7 d	0.96
		0 d	7 d	0.89
	0.25	0 d	3 d	< 0.001
		7 d	3 d	0.13
		0 d	7 d	0.01
	0.50	0 d	3 d	< 0.001
		7 d	3 d	0.24
		0 d	7 d	0.01
	0.75	0 d	3 d	< 0.0001
		7 d	3 d	0.78
		0 d	7 d	< 0.0001
	1.00	0 d	3 d	< 0.0001
		3 d	7 d	0.85
		0 d	7 d	< 0.0001
	1.25	0 d	3 d	< 0.0001
		3 d	7 d	0.81
		0 d	7 d	< 0.0001
	1.50	0 d	3 d	< 0.0001
		7 d	3 d	0.37
		0 d	7 d	< 0.0001
CD8 ( +)	0.10	0 d	3 d	0.71
		3 d	7 d	0.99
		0 d	7 d	0.71
	0.25	0 d	3 d	< 0.01
		7 d	3 d	0.25
		0 d	7 d	0.03
	0.50	0 d	3 d	< 0.01
		7 d	3 d	0.30
		0 d	7 d	0.03
	0.75	0 d	3 d	< 0.0001
		7 d	3 d	0.42
		0 d	7 d	< 0.001
	1.00	0 d	3 d	< 0.001
		3 d	7 d	0.82
		0 d	7 d	< 0.001
	1.25	0 d	3 d	< 0.0001
		3 d	7 d	0.72
		0 d	7 d	< 0.0001
	1.50	0 d	3 d	< 0.0001
		7 d	3 d	0.89
		0 d	7 d	< 0.0001
B220 ( +)	0.10	0 d	3 d	0.18
		3 d	7 d	0.27
		0 d	7 d	0.02 *
	0.25	0 d	3 d	< 0.0001
		3 d	7 d	0.06
		0 d	7 d	< 0.0001
	0.50	0 d	3 d	< 0.01
		3 d	7 d	0.89
		0 d	7 d	< 0.01
	0.75	0 d	3 d	< 0.0001
		7 d	3 d	0.71
		0 d	7 d	< 0.001
	1.00	0 d	3 d	< 0.001
		3 d	7 d	0.97
		0 d	7 d	< 0.001
	1.25	0 d	3 d	< 0.001
		3 d	7 d	0.47
		0 d	7 d	< 0.001
	1.50	0 d	3 d	< 0.0001
		7 d	3 d	0.61
		0 d	7 d	< 0.001

*The significant difference between day 0 and day 7 at a dose of 0.10 Gy was observed exclusively in the B220(+) lymphocytes. No significant differences were noted between day 0 and day 7 at a dose of 0.10 Gy in other lymphocyte subtypes.

The internal irradiation (D_blood_ = 0.16 Gy) experiments demonstrated that the formation of γ-H2AX was reversed within 24 h. The MFI’/MFI ratios of the experimental and control groups did not differ significantly after 24 h. The t-test results are shown in Table 4.

**Table 4:** t-Test results of internal exposure experiments.

Cells	Level	−Level	*P*-value
CD4 ( +)	1 h	Control	0.02
	24 h	Control	0.85
	1 h	24 h	0.02
CD8 ( +)	1 h	Control	< 0.001
	24 h	Control	0.76
	1 h	24 h	0.01
B220 ( +)	1 h	Control	< 0.0001
	24 h	Control	0.54
	1 h	24 h	< 0.0001

Meanwhile, the absorbed doses represented in the percentages of accumulated radioactivity to the injected dose per gram gradually decreased to a level close to the controls with time. The specific details are shown in Table 5.

**Table 5:** Percentages of accumulated radioactivity to the injected dose per gram (%ID/g) were used to measure absorbed doses.

Time after irradiation (h)	Experiment (%ID/g)
1	6.12 ± 2.60
3	2.54 ± 0.61
24	0.05 ± 0.02

## Discussion and conclusion

The present study proposed an innovative evaluation method of IR-induced DNA damage that quantitatively presents damage levels through γ-H2AX foci flow cytometry analyses. In contrast to previous studies^4,16–19^ which have primarily focused on the examination of either the dose dependence or the temporal alternation of radiation damage or the examination of one aspect of external or internal irradiation, this study confirmed the dose and time dependence of radiation damage caused by both internal and external irradiation under consistent laboratory conditions.

Various methods are available for detecting DSB, each with distinct characteristics that suit different purposes. Pulsed-field gel electrophoresis is the most commonly used method in analyzing DSBs; however, it has limitations in monitoring persisting DSBs^20^. Microscopic observation of the γ-H2AX level is another commonly used method; however, it can be cumbersome and time-consuming. In comparison, FCM allows for a rapid, sensitive, and quantitative evaluation of DSBs^5,21^. Notably, γ-H2AX flow cytometry analyses were considered inadequate for detecting DNA damage induced by low-dose irradiation^14^. This study found that exposure to a radiation dose of ≤ 0.10 Gy resulted in no significant alteration in γ-H2AX levels, suggesting that FCM may be suitable for assessing the presence of detectable damage in radiation-exposed individuals and estimating the absorbed blood dose in a single-point test.

In the study, we found that DNA damage in lymphocytes is linearly positively correlated with the irradiation doses of external radiation received by mice. The results further revealed that the CD45R/B220 lymphocyte subset is relatively more sensitive to radiation^7^, as indicated by the equation in Table 2. However, linear regression analyses of the in vivo internal irradiation experiment were not conducted due to the high-dose exposure to the blood of external irradiation in mice compared to the low doses of radiopharmaceuticals injected in the mice. This is because internal irradiation does not result in a higher radiation dose as the radioisotope transfer from the blood is completed quickly after administration. Considering that the degree of DSB repair varies depending on the radiation type and the damage increases with the radiation dose, it is conceivable that similar linearity could be observed in internal irradiation. Due to ethical reasons, high-dose administration to the mice was precluded. When evaluating DNA damage induced by internal radiation exposure to blood with FCM, it is challenging to detect low-dose-induced DSBs, and the accuracy of linearity is expected to vary with the evaluation time.

The study first investigated β-ray-induced DSB in mice. IR-induced DNA damage assessment through the FCM method has been reported^10,22^; however, radiation emitted by the Lu-177 or Ra-223 nuclide-conjugated radioactive pharmaceuticals is mainly α-rays^18,22^, not the β-rays emitted by I-131 that we used in this study. Besides, Lenka Zárybnická et al. reported that H2AX phosphorylation of lymphocyte subsets in response to in vivo irradiation in rats had the potential to be applied to bio-dosimetry^5^; however, related studies in mice had never been reported before, which highlights the need for exploring the utility of H2AX phosphorylation as a biomarker and further research to investigate biodosimetry in mice.

Due to the technical reason that if the same mouse is blood-collected twice or more within 3 days, severe anemia or the death of the experimental individual will occur, leading to deviations in results, blood samples between 0 and 3 days were not included in our study. The results of the present animal experiment also showed that there was no significant difference in the level of γ-H2AX 3 days and 7 days after irradiation. A relevant clinical study reported that DNA repair detected in mononuclear cells has been almost completed 24 h after receiving γ or X-rays^19^. A Bayesian model to predict individual radiosensitivity in patients also reported that the number of DSB foci is positively correlated with the doses^23^, and the focus count was expected to enter a stable state in 24 h. As Schumann et al. reported no data after 24 h and significantly higher average numbers of foci per cell after 24 h than the baseline, it was reasonable to conclude that the DNA damage repair may be complete within three days, and more data were required between the time points of 24 h and 72 h.

Although the colocalization of γ-H2AX/53BP1 is currently considered the most reliable marker of DSB, considering the dephosphorylation of γ-H2AX was reported to represent recovery from DNA damage, γ-H2AX foci analyses proved to be efficient enough in providing quantitative DSB evaluations^16,24^. Additionally, the 53BP1 protein is not induced by radiation but instead undergoes relocalization to the sites of double-strand breaks (DSBs). Consequently, this molecular marker cannot be utilized for the detection of DSBs through conventional flow cytometry (FCM) methods^4,25^. Imaging Flow Cytometry (IFC) was reported to be able to perform the colocalization of γ-H2AX/53BP1^4^, but the instrument and analysis system required were still not used extensively throughout the world. The present study also showed that the FCM method is a valid method for the quantitative evaluation of IR-induced DNA damage in radiosensitive lymphocytes.

In conclusion, DNA damage in lymphocytes caused by external γ-ray exposure damage was proved to be positively dependent on the absorbed radiation dose. The recovery from IR-induced damage was confirmed to be less than 3 days in the present study, while low-dose internal irradiation caused damage recuperated relatively rapidly in 24 h. Our study demonstrated that the FCM method could quantitatively assess IR-induced lymphocyte damage rapidly and effectively.

## Methods

### Animals

Wild-type mice (BALB/cCrSlc) were obtained from the Jackson Laboratory and bred in-house. Male mice aged 8–12 weeks old and weighing 23.0–25.0 g were included in the study. Each experimental group consisted of six mice. Peripheral venous blood was collected into the heparin-containing solution using the experimental siphon tubes (final concentration 100 International Unit (UI)/ml, Mochida Pharmaceutical Co., LTD., Tokyo, Japan). The Ethics Committee of Kanazawa University approved the study (AP-184027), and the study is conducted and reported in compliance with the national legislation and the ARRIVE guidelines. Euthanasia via carbon dioxide inhalation was performed after the experiment as a humane method of animal sacrifice.

### Irradiation and peripheral blood collection

Ionizing irradiation was provided using an X-ray Generator System (MBR-1520R-3; Hitachi Power Solutions Co. Ltd., Ibaraki, Japan). Before X-ray exposure, air kerma rate measurements were performed. The dose rate at the location where the subject is placed and the dose rate at the monitoring location for accumulated dose during irradiation were premeasured. The dose administered to the subject was then automatically calculated based on the ratio between these two rates, and irradiation is automatically stopped. Mice were anesthetized with isoflurane prior to blood collection. The volume of each blood sample was 500 μL.

### External irradiation experiment: in vitro

For FCM, the peripheral venous blood samples of eight experimental groups (6 mice/group) collected were exposed to the following irradiation doses: 0.10, 0.25, 0.75, 1.00, 1.25, 1.50, 1.75, and 2.00 Gy (tube voltage, 150 kV; tube current, 20 mA; filter, 0.5 mm Al and 0.2 mm Cu; distance between focus and target, 320 mm). Blood samples excluded from irradiation were set as controls. For immunohistochemistry (IHC) staining, one experimental group (3 mice/group) were irradiated with doses of 1 Gy and one control group of 3 mice were set for observation and analyses.

### External irradiation experiment: in vivo

The mice of eight experimental groups (6 mice/group) received whole-body irradiation at doses of 0.10, 0.25, 0.5, 0.75, 1.00, 1.25, 1.5, 2.00 Gy, respectively for each observation time point (tube voltage, 150 kV; tube current, 15 mA; filter, 0.5 mm Al and 0.5 mm Cu; distance between focus and target, 400 mm). During irradiation, mice were confined to a small cage (fan-shaped; radius 10 cm, height 4.5 cm, angle 30°) specific for X-irradiation. Nonirradiated animals served as controls. Blood samples were collected on day 0, day 3, day 7, and day 14 after radiation exposure.

### Internal irradiation

Fourteen mice were injected with I-131 sodium iodide solution (74 MBq) intraperitoneally (five mice/group), and four mice injected with stroke-physiological saline solution were set as the control group. Blood samples were collected at the time points of 1 h and 24 h by group (5 mice/group).

### Sample preparation

Collected peripheral blood samples were lysed using red blood cell lysis reagents (RBC Lysis Buffer). Each sample was divided into two aliquots of 50 μl each at a density of 1 × 10^6^ cells/50 μl for the following T- and B-cell immunofluorescence staining. Peripheral blood mononuclear cells for IHC staining were fixed by adding 4% Paraformaldehyde Phosphate Buffer Solution at room temperature after 3 h incubation in 12-well plates (37 °C, 5% CO_2_).

### Immunofluorescence staining and evaluation of DNA damage

The following monoclonal antibodies (mAbs) were purchased from BioLegend, BD Bioscience and Abcam: antimouse CD8a antibody (clone 53–6.7, PE/Cyanine7 conjugated), antimouse CD4 antibody (clone GK1.5, FITC conjugated), antimouse CD45R/B220 antibody (clone RA3-6B2, FITC conjugated), anti-H2AX (pS139) antibody (clone N1-431, PE-conjugated) and anti-gamma H2AX (pS139) antibody (clone EP854(2)Y, Alexa Fluor® 488-conjugated). Cell surface antigen staining was performed for FCM only. Subsequently, we used a fixation and permeabilization solution to permeate the membrane and conduct intracytoplasmic staining using the aforementioned anti-H2AX (pS139) antibody. The whole staining procedure was conducted per the manufacturer's guidelines and protocols. Stained cells were run on a flow cytometer (RF-500, Sysmex Corporation, Kobe, Japan) for FCM.

### Absorbed dose measurement

Radioactivity measurements (percentage of injected dose [%ID] per gram) were performed at 1, 3, and 24 h after injection using an autowell gamma system (AccuFLEX γ ARC-800, Hitachi Aloka Medical, Ltd., Mitaka, Tokyo) in the internal irradiation experiment. All data during the 24-h washout were used to plot a blood time-exposure curve with a double-exponential curve fit. Nonlinear regression analyses were performed using MagicPlot Pro software (ver 2.7.2) to obtain exponential time constants and coefficients. Circulating Blood Volumes of Mice were calculated (72 ml/kg). Then, the blood accumulated activity, Ã_blood_, was derived. The blood dose was calculated using the medical internal radiation dose formula:

$${\text{D}}_{{{\text{blood}}}} = \tilde{A}_{{{\text{blood}}}} {\text{S}}_{{{\text{blood}} \leftarrow {\text{blood}}}}$$

where D_blood_ is the mean absorbed dose, and S_blood←blood_ ($3.18\times {10}^{-11} \, \mathrm{Gy } \, {{\text{s}}}^{-1} \, {{\text{Bq}}}^{-1} \, {\text{ml}}$) is the blood-to-blood *S-*factor for I-131^19^.

### Data analyses and statistics

All the flow cytometry data were analyzed using FlowJo software (Version 10.8.1, Becton Dickinson & Company (BD), Franklin Lakes, New Jersey, USA). All results are presented as the mean ± standard deviation (SD)/standard error. The significance of differences between groups was assessed using the one-way analysis of variance and t-tests. The radioactive correlation linear fit was performed on both external and internal irradiation experiment results. A *P*-value of < 0.05 was considered statistically significant. All the statistical analyses were performed using JMP Pro software (version 16.0.0, SAS Institute, Cary, NC, USA).

## Data Availability

All the data that constitute this study are available from the corresponding author on request.
